# Squeeze-Film Air Damping of a Five-Axis Electrostatic Bearing for Rotary Micromotors

**DOI:** 10.3390/s17051119

**Published:** 2017-05-13

**Authors:** Shunyue Wang, Fengtian Han, Boqian Sun, Haixia Li

**Affiliations:** Department of Precision Instrument, Tsinghua University, Beijing 100084, China; wsy15@mails.tsinghua.edu.cn (S.W.); sbq12@mails.tsinghua.edu.cn (B.S.)

**Keywords:** squeeze-film damping, finite-element method, multi-physics simulation, electric bearing, MEMS micromotor, air-film damping measurement

## Abstract

Air-film damping, which dominates over other losses, plays a significant role in the dynamic response of many micro-fabricated devices with a movable mass suspended by various bearing mechanisms. Modeling the damping characteristics accurately will be greatly helpful to the bearing design, control, and test in various micromotor devices. This paper presents the simulated and experimental squeeze-film air damping results of an electrostatic bearing for use in a rotary high-speed micromotor. It is shown that the boundary condition to solve the three-dimensional Reynolds equation, which governs the squeeze-film damping in the air gap between the rotor and its surrounding stator sealed in a three-layer evacuated cavity, behaves with strong cross-axis coupling characteristics. To accurately characterize the damping effect, a set of multiphysics finite-element simulations are performed by computing both the rotor velocity and the distribution of the viscous damping force acting on the rotor. The damping characteristics varying with several key structure parameters are simulated and discussed to optimize the device structure for desirable rotor dynamics. An electrical measurement method is also proposed and applied to validate the numerical results of the damping coefficients experimentally. Given that the frequency response of the electric bearing is critically dependent on the damping coefficients at atmospheric pressure, a solution to the air-film damping measurement problem is presented by taking approximate curve fitting of multi-axis experimental frequency responses. The measured squeeze-film damping coefficients for the five-axis electric bearing agrees well with the numerical solutions. This indicates that numerical multiphysics simulation is an effective method to accurately examine the air-film damping effect for complex device geometry and arbitrary boundary condition. The accurate damping coefficients obtained by FEM simulation will greatly simplify the design of the five-axis bearing control system and facilitate the initial suspension test of the rotor for various micromotor devices.

## 1. Introduction

Various types of microfabricated motors have been successfully designed, fabricated, and tested for microsensors and microactuators [1,2,3]. The rotor of a rotary micromotor is typically supported on a mechanically attached bearing using silicon microfabrication technology, the contact friction and wear between the surfaces of a micro-scale rotor and its bearing are typically large during the rotation operation [4,5]. Hence, inherent friction and wear caused by the mechanical micro-bearing greatly restricts the lifetime and efficiency of traditional micromotors. In order to minimize the mechanical friction and wear, various miniaturized bearing mechanisms, such as contactless electrostatic and electromagnetic [2,6], liquid-suspended [7] and gas-lubricated bearings [8], have been reported since the early 1990s. Among them, active electrostatic bearings are ideally suited for rotary micromachined devices as they are comparatively compatible with existing microfabrication technology and capable of integrating bearing control electronics into the same chip to realize low-voltage and low-power operation [9,10,11]. 

Currently, several electrostatic bearing systems were developed as an effective solution to the problem of friction and wear in traditional micro-electro-mechanical system (MEMS) micromotors. Most of the electrostatic bearing systems are designed for application of high-speed spinning-rotor gyroscopes or multi-axis force-balanced accelerometers [2,12,13,14,15,16]. The rotor is commonly sealed within a triple-layer structure and surrounded by stator electrodes for suspension and rotation control. Several device designs with ring-, disk-, and sphere-shaped rotors suspended electrostatically in the center of their stator cavities were reported in recent years. Although vacuum packaging of these MEMS devices are required for rotor-spinning gyroscopes in order to minimize the viscous drag effect and yield much higher spin speed [2,12], lots of preliminary device tests at atmospheric pressure are necessary to verify its function and performance and screen out any unqualified device before being vacuum packaged. Moreover, electrostatically suspended MEMS accelerometers are mostly operated directly at atmospheric pressure to achieve low cost [14]. Considering the electrostatic suspension is inherent open-loop unstable and five suspension degrees of freedom (DOFs) are required to support a ring-shaped rotor, design and test of these multi-axis electrostatic suspension loops are challenging due to relatively large modeling error. Our early work reported a variable-capacitance micromotor utilizing a five-DOF electrostatic bearing as a means of providing contactless suspension of the rotor [10]. However, initial suspension control and test of the five-axis electric bearing system are usually time consuming and labor intensive at atmospheric pressure due to unknown air-film damping coefficients. Accurate modeling of the air-film damping is not only helpful to facilitate successful suspension of multi-axis electric bearings, but also necessary to optimize the device’s geometry and dimensions by considering the bearing requirements on desirable dynamic response and stability. Generally, the model describing the squeeze damping force may be generated by two different approaches, i.e., analytical approximation and numerical modeling using finite-element method (FEM) simulation. 

Currently, the study of air-film damping for various MEMS devices focuses mainly on simple geometries and unsealed two-layer structures [17,18,19,20]. Moreover, most of the published work on electrostatic bearings focused on device fabrication techniques, control, and test of electrostatic bearings and vacuum spinning-rotor gyroscopes [2,9,10,11,12,13,14,15,16]. However, little work has been reported on the air-film damping modeling and test of these electrostatically suspended devices in which the rotor is sealed in a three-layer evacuated cavity. A finite-element model via an analogy to heat transfer theory was presented in [21] to extract squeeze film damping coefficients for transverse and rotational motion of a disk-shaper rotor. However, the FEM solutions are based on a simplified, unsealed three-layer structure by removing radial electrodes, which is totally different from the sealed structure due to their difference in boundary pressure conditions. Previous work by the authors presented an analytical air-film damping model which is also based on an unsealed three-layer structure and simplified boundary conditions [22]. It was found that the analytical results are not in good agreement with experimental suspension responses and lots of trial and error have been made before successfully initial suspension of the rotor. Accurate measurements and modeling of the described damping effects are a challenge for the designer and are not supported by consolidated experiments reported in the literature at present.

This paper presents a numerical solution of the squeeze-film damping coefficients for a five-axis electrostatic bearing system operated at atmospheric pressure. The FEM-based multiphysics simulation is described as a design example to model accurately the damping effect of the sealed triple-layer device with complex geometry. The damping characteristics varying with several key structure parameters are simulated and discussed to optimize the device structure for desirable suspension responses. An electrical measurement method is also proposed and has validated the numerical results of the damping coefficients experimentally. The accurate damping coefficients obtained by FEM simulation can be used to model the bearing dynamics and facilitate the initial suspension and test of various micromotor devices.

## 2. Description of the Micro-Motor

### 2.1. Device Geometry

**Figure 1:**
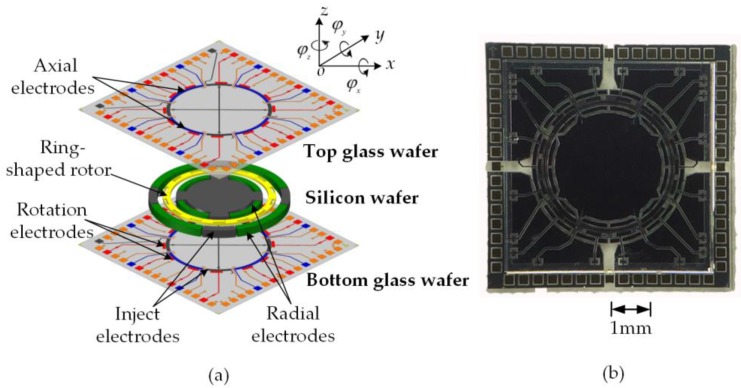
Micromachined electrostatic motor integrated with a five-axis electrostatic bearing: (**a**) exploded view of the device with a glass/silicon/glass bonded triple-layer structure and (**b**) a close-up view of the fabricated device.

A typical device structure of the micro-motor consists of two Pyrex 7740 glass wafers and one silicon wafer, which is designed with a glass/silicon/glass triple-layer bonding structure and a complex electrode pattern as depicted in Figure 1a. The essential geometry comprises a spinning rotor, an electric bearing that supports the rotor at its null position, and a variable-capacitance motor that drives the levitated rotor at certain speed. Stator electrodes are symmetrically arranged around the rotor to form arrays of truncated pie-shaped electrodes for capacitive position sensing and electrostatic actuation. Planar electrodes located on the top and bottom glass wafers are used for axial suspension in three DOFs: out-of-plane translation, *z*, and rotations around in-plane axes, *φ_x_* and *φ_y_*. The outmost electrodes on each glass wafers are used for rotor spin drive operated in a planar variable-capacitance motor. The intermediate silicon layer forms the ring-shaped rotor, inner and outer radial electrodes used for suspension control along the *x*- and *y*-axes. The micromotor device was fabricated with bulk micromachining based on silicon on glass (SOG) technique [23]. The main design parameters of the device geometry are listed in Table 1. The size of the fabricated device is 6.5 mm × 6.5 mm × 1.1 mm, as depicted in Figure 1b.

### 2.2. Air-Film Damping

In contrast to conventional micromotors where the friction completely dominates the dynamic behavior of the motor, the viscous damping effect produced by the surrounding air plays a critical role in energy dissipation and attainable rotor speed of the levitated motor. A dynamic model of the variable-capacitance micromotor operated in atmospheric environment was presented using a simplified damping model and validated experimentally [23]. On the other hand, air-film damping effect also plays an important role on the dynamic response and stability of the five-axis electric bearing. Proper air-film damping design will provide desirable stability and dynamic response. Compared with slide-film damping, squeeze-film damping has dominant effects on the bearing dynamics [22]. In this work, we will focus on modeling, simulation, and testing of the squeeze-film air damping characteristics.

### 2.3. Electrostatic Suspension

Basically, the rotor is a free-spinning ring suspended and centered in an evacuated cavity with forces produced by a five-axis electrostatic suspension system for use as an electric microbearing. To maintain stable suspension, the rotor is actively suspended in five DOFs: translations along the *x*-, *y*-, and *z*-directions, and rotations around two in-plane axes. If the cross-coupling effects among the different axes are ignored, the dynamics of the rotor can be modeled by five uncoupled 1-DOF systems [10].

{(1a)me¨i+bie˙i=Fi(1b)Jα¨j+bjα˙j=Mj

where *m* and *J* are the mass and moment of inertia of the rotor, *e* and *α* are the linear and angular displacements of the rotor away from its equilibrium position, *b* is the air-film damping coefficient, *F* and *M* are the electrostatic feedback force and torque, the subscript *i* = *x*, *y*, *z* denotes the axis along which the force is produced, and *j* = *φ_x_*, *φ_y_* denotes the axis around which the torque is produced, respectively.

When small displacements of the rotor occur around its equilibrium position, the electrostatic force produced by the charged set of the suspension electrodes on the rotor along the considered *x*-axis can be linearized as

(2) $$F_{x}=K_{v}V_{x}+K_{x}x$$

where *K*_v_ and *K_x_* are the electrostatic actuator gain and the position stiffness, respectively.

Substituting Equation (2) into Equation (1a) and taking the Laplace transform yields the dynamics of the rotor:

(3) $$\frac{X(s)}{V_{x}(s)}=\frac{K_{v}}{ms^{2}+b_{x}s-K_{x}}$$

The dynamics of the rotor in four other DOFs can be found by substituting appropriate variables in Equation (3). Given design parameters of the electric bearing system, the model parameters *m*, *K*_v_, and *K_x_* can be obtained analytically [10]. However, the damping coefficient *b_x_* is hard to be computed accurately by an analytical model as the air-film damping characteristics behaves strong cross-axis coupling for the rotor motion constrained inside a sealed cavity. This suggests that experimental measurements or FEM simulations could be utilized to determine the air-film damping coefficient.

**Figure 2:**
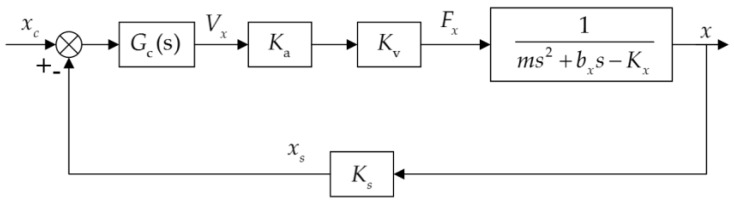
Block diagram of the closed-loop suspension control loop, one degree of freedom of five.

Equation (3) also shows that the electrostatic bearing can be modeled as a mechanical mass-spring-damper system. The levitated rotor is modeled as a mass mechanically attached to a rigid frame through an electrical spring and an air-film damper, despite the absence of a physical spring connecting the rotor to the substrate. By inspecting the characteristic equation of the transfer function in Equation (3), open-loop instability due to the negative position stiffness, i.e., −*K_x_*, can be found as there is a pole on the right-hand side of the *s*-plane. Consequently, active feedback control must be utilized to stabilize the suspension servo. A simplified block diagram of the closed-loop bearing control loop is shown in Figure 2, where *K*_s_, *K*_a_, and *G*_c_(*s*) are the sensitivity of the rotor position sensor, gain of the voltage amplifier, and transfer function of the feedback controller, respectively.

The closed-loop transfer function from the position command input, *x*_c_, to position sensor output, *x*_s_, can be used as a measure of the loop dynamic performance and is given by

(4) $$G_{\mathrm{cl}}(s)=\frac{X_{s}(s)}{X_{c}(s)}=\frac{K_{v}K_{s}K_{a}G_{c}(s)}{ms^{2}+b_{x}s-K_{x}+K_{v}K_{s}K_{a}G_{c}(s)}$$

As the closed-loop system can be maintained stably by proper design of feedback controller, it is possible to obtain the damping coefficient experimentally from measured frequency response. Considering all parameters except for *b_x_* can be obtained analytically, then we could extract *b_x_* by accurately fitting a sweeping-frequency measurement with its simulated frequency response curve. The air-film damping in four other DOFs can be extracted in a similar way by measuring their frequency responses respectively. Using this electrical measurement method, we can verify the simulated results of five-DOF squeeze-film damping by experimental measurements, as detailed procedure description presented in Section 5.

## 3. Reynolds Equation for Squeeze-Film Damping

**Figure 3:**
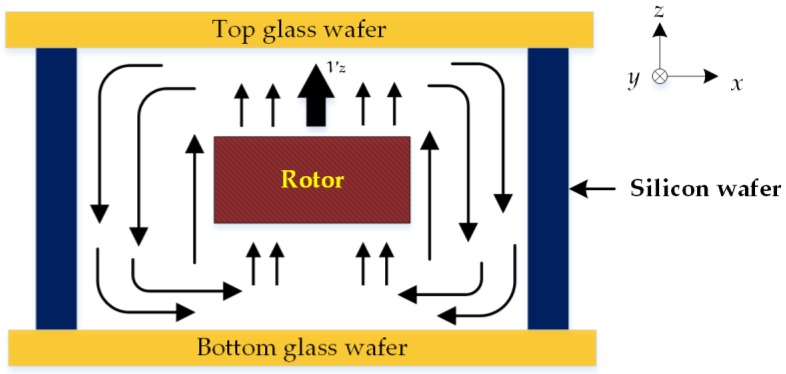
Cross-sectional view of the air flow in the sealed glass/silicon/glass triple-layer cavity.

The triple-layer bonded cavity used in modeling the squeeze-film air damping consists of the top and bottom glass wafers, and middle silicon layer, as depicted in Figure 1a. When the suspended rotor moves upward with a velocity *ν_z_*, the resistive force to the rotor moving normally against the top/bottom glass wafers is caused by the viscous flow of air between the rotor and surrounded stator. A cross-sectional view of the air flow is shown in Figure 3.

The damping pressure consists of two main components: the component to cause the viscous flow of gas when the air is squeezed out of or sucked into the rotor-stator gap and that to cause the compression of the air film [24]. If the air film oscillating inside the cavity at low frequency satisfies incompressible condition, the linearized Reynolds equation governing three-dimensional (3D) air film characteristics is [25,26,27]

(5) $$\frac{\partial^{2}p}{\partial x^{2}}+\frac{\partial^{2}p}{\partial y^{2}}+\frac{\partial^{2}p}{\partial z^{2}}=\frac{12\mu }{d^{3}}\frac{\partial d}{\partial t}$$

where *p* is the damping pressure in the air film, *d* is the thickness of the film, and *μ* is the coefficient of viscosity of the fluid.

Generally, the analytical model could be solved for simple geometry with trivial boundary conditions, i.e., the air at the borders is at ambient pressure. Using similar assumption on boundary conditions, the analytical solutions of squeeze-film damping for the five-axis electric bearing have been derived in [22]. However, in practical micromotor devices, the air flow escaping from the borders might significantly increases the viscous damping force. In order to include the border effects on the analytical model, an effective device size is introduced in [25]. For a two-dimensional plate, FEM simulations method was used to predict the values of the effective device size, e.g., length and width. In this way, the damping force for the enlarged plate with trivial boundary conditions has the same value as the damping force of the real device size considering the border effects [25].

Naturally, the border effect depends on the geometry of the gas outlet region. As the sealed device illustrated in Figure 3, the moveable rotor is suspended in the center of the evacuated cavity and has six DOFs of motion. As the air flow generated by one DOF could affect the pressure distribution of other DOFs, the realistic boundary condition for Equation (5) shows strong cross-axis coupling characteristics and is too complex to solve analytically. Generally, analytical solutions are inherently limited to simple geometries and numerical approaches are required to model more complex device structures [21]. Next, we will focus on how to obtain a numerical solution of the squeeze-film damping using multiphysics FEM simulations.

## 4. Numerical Multiphysics Simulations

Squeeze-film damping is the main source of energy dissipation affecting the dynamic behavior of most MEMS devices. It is mainly determined by the degree of air rarefaction and feature size of the air film. In this work, multiphysics FEM simulations for the structure are performed to determine the air-film damping coefficients [28,29]. The air surrounding movable rotor is responsible for five-axis coupled fluid-structure interaction that changes the bearing dynamics.

### 4.1. Gas Compressibility and Rarefaction

Gas flowage in the cavity can be characterized by the Knudsen number

(6) $$K_{n}=\frac{\lambda }{d}$$

where *λ* is the mean free path of the molecules, and *d* is the gas film depth in normal direction of gas surface [20,25].

Given *d* = 6 μm and *λ =* 70 nm, the resulted *K*_n_ is 0.01167 for the electrostatic bearing. Hence, the gas flowage between the suspended rotor and associated stationary stators can be regarded as laminar flow [22].

Compression coefficient *σ* is given to evaluate gas film compressibility

(7) $$\sigma =\frac{12\mu_{\mathrm{eff}}l^{2}}{d^{2}Pa}\omega$$

The effective viscosity of air *μ*_eff_ is given by a simple, empirical expression reported by Veijola et al. [30].

(8) $$\mu_{\mathrm{eff}}=\frac{\mu }{1+9.638K_{n}^{1.159}}$$

where *l* is the characteristic length and *ω* is the gas film vibration frequency.

When *σ* << 1, the gas film is incompressible and damping effect in the air film is dominant [22]. Under a certain condition of characteristic size and environment pressure, *σ* is solely determined by *ω*. Assuming the operating frequency of electric microbearing is typically less than 500 Hz, the corresponding *σ* is lower than 0.181. This indicates the air film satisfies the incompressible condition [22]. 

### 4.2. Simulation Results 

To characterize as accurately as possible the squeeze-film damping coefficients in the sealed structure, FEM-based multiphysics simulations are performed to obtain numerical solutions. A commercial finite-element software (Comsol) is applied to solve the 3D incompressible flow model. The nominal device dimensions listed in Table 1 are used in the following simulations. Several material parameters of the silicon rotor used are as follows: Young’s modulus E = 170 GPa, Poisson’s ratio ν = 0.28, and density ρ = 2329 kg/m^3^. 

The FEM software can automatically generate a 3D mesh, consisting of tetrahedral elements modeling the moving rotor, the fluid surrounding the rotor and electrodes, etc. Generally, the volume of fluid surrounding the rotor should be considered in the finite-element analysis (FEA), which makes complete 3D models computationally very heavy [18]. A mesh of 10^6^ elements or more are attempted to reach a compromise between numerical precision and computation efficiency. Simulation results with a larger number of elements (∼1.5 × 10^6^) indicate that the relative change in simulated squeeze-film damping coefficients is below 1%. Hence, the mesh number is approximately set at 10^6^ elements in following simulations. 

Assuming that the rotor moves at a non-zero but small velocity along the radial or axial direction at standard atmospheric pressure (1 atm) and temperature 300 K, a three-dimensional incompressible Navier–Stokes equation was solved for the air film between the rotor and the surrounded stators. The advantage of using the finite-element method lies in the ability to model arbitrary device geometries and boundary conditions. In the case where the rotor moves normally with respect to the top glass wafer along the *z*-axis, the squeeze film damping coefficient is evaluated as [20]

(9) $$b_{z}=\frac{\int p(x,y)dxdy}{v_{z}}$$

where *p*(*x*,*y*) is the pressure field over the moving rotor.

**Figure 4:**
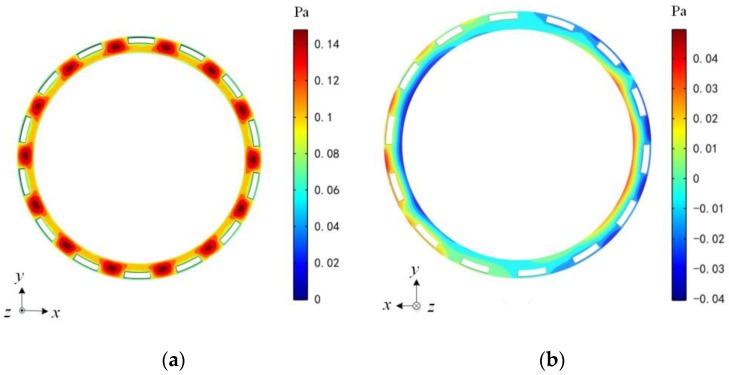
Pressure distribution in the air gap caused by the movement of the rotor (**a**) translation along the *z*-direction and (**b**) translation along the *x*-direction.

The pressure distribution caused by the movement of the rotor along the *z*-axis is shown in Figure 4a. Clearly, the maximum pressure distributes uniformly along 14 outmost slots. Using Equation (9), the coefficient of the damping acted on the annular rotor by a 6 μm squeezed air film thickness is 2.41 × 10^−2^ N/(m·s^−1^). It seems that the inclusion of these motor slots in the rotor greatly reduces the squeeze-film damping coefficients of the axial motion, as described in such perforated MEMS structures [25].

FEM-based damping simulations for the motion of the rotor along two radial *x*- and *y*-axes are also performed. Figure 4b shows the pressure distribution of air flow as the rotor moves along the *x* axis. Using the structure parameters listed in Table 1, the numerical solutions of the radial squeeze-film damping coefficients are *b_x_* = *b_y_* = 2.81 × 10^−3^ N/(m·s^−1^).

**Figure 5:**
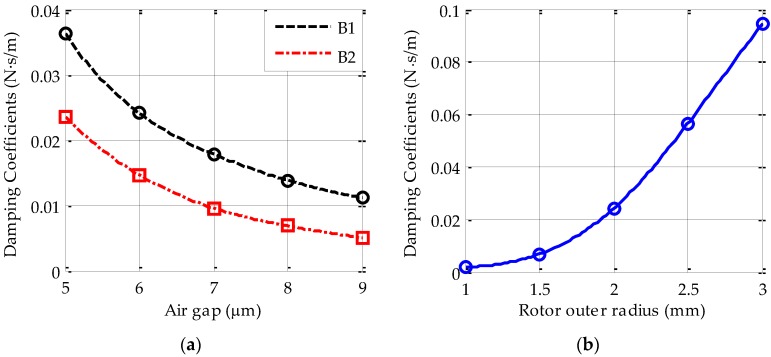
The simulated *z*-axis damping coefficient (**a**) damping coefficient varying with the axial gap for the sealed (B1) and the unsealed (B2) devices; (**b**) damping coefficient varying with the rotor outer radius for the sealed device.

The nominal rotor-stator gap is one of the crucial design parameters in optimization of the overall device structure. Figure 5a shows the *z*-axis squeeze-film damping coefficients as a function of the axial air gap varying from 5 μm to 9 μm. It is clear that the damping effect decreases gradually as the gap increases.

For comparison, Figure 5a shows the numerical damping coefficients for the sealed device structure (B1) and an unsealed one (B2), i.e., all radial electrodes formed by the intermediate silicon layer are removed during the *z*-axis simulation. Although the two curves show virtually the same change trend with increasing gap, the sealed device shows steadily a much larger value than the unsealed one. This difference can be explained by air coupling effect, which has been considered in the sealed device but is neglected in the unsealed structure. Additionally, the simulation results also show that the curve B1 can be fitted by a quadratic form of the axial gap *d_a_*, i.e., *b_z_* = 0.9884 × *d*_a_^−2^.

Furthermore, the squeeze-film damping coefficient varying with the rotor outer radius is simulated and shown in Figure 5b, where a constant ratio of the inner rotor radius to its outer radius is set at 0.865. The FEM results show the squeeze-film damping rises sharply by increasing the rotor outer radius. The fitted curve in Figure 5b can be approximated to *b_z_* = 0.0026 × *r*_0_^3^. Considering both the rotor mass and the electrostatic actuator gain are proportional to the square of the rotor radius, it is clear that the dynamic behavior of an electric bearing with a large rotor size will be limited more by air damping constraints.

**Figure 6:**
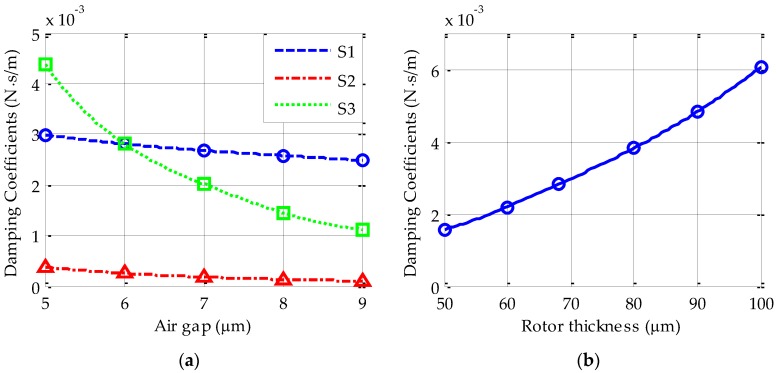
The simulated *x*-axis damping coefficient (**a**) damping coefficient varying with the radial (S1, S2) or axial (S3) rotor-stator gap and (**b**) damping coefficient varying with the thickness of the rotor for the sealed device. Note that the curves S1 and S3 are obtained by assuming a sealed device while the curve S2 is simulated from an unsealed one.

Figure 6a shows three simulated curves of the *x*-axis damping-gap characteristics for comparison. Note that the curves S1 and S3 are obtained by assuming a sealed device while the curve S2 is simulated from an unsealed one, i.e., the top and bottom glass wafers are removed during the *x*-axis simulation. In order to investigate the air flow coupling effect, the radial (*x*/*y*) damping coefficients are simulated by setting a varying radial gap (S1) and axial gap (S3), respectively. The curve S1 indicates that the radial damping coefficient reduces slightly when the radial gap increases from 5 μm to 9 μm. However, the curve S3 shows that the radial damping is more sensitive to change of the axial gap. It indicates that the damping couple of the sealed air film has a more significant effect on the *x*-axis than the *z*-axis. This phenomenon can be explained by examining the rotor geometry with a thickness of only 68 μm and an outer radius up to 2 mm. 

On the other hand, as mentioned above, coupling effect does not exist in the unsealed device, this implies that much different damping characteristics will be exhibited between a sealed device and an unsealed one. This can be verified when comparing the curve S1 with that of S2. It is clear that the curve S1 shows much larger squeeze-film damping than the curve S2. For instance, the FEM damping coefficients by setting a radial gap of 6 μm are 2.81 × 10^−3^ N/(m·s^−1^) for the sealed device and 2.53 × 10^−4^ N/(m·s^−1^) for the unsealed one. This suggests that the simplified unsealed geometry is not suited to accurately modelling the air-film damping because significant coupling effect in actual devices is ignored.

The change of the squeeze-film damping coefficient as a function of the thickness of rotor is also investigated. Figure 6b shows the simulated results by setting the rotor thickness ranging from 50 to 100 μm. The curve indicates that the squeeze-film damping coefficient rises rapidly when increasing the rotor thickness.

From the simulations results shown above, it is clear that the geometry parameters of the annular rotor has significant effects on the radial and axial squeeze-film damping coefficients. Generally speaking, the damping of the suspended rotor can be reduced by increasing the air film thickness along the direction of transverse motion or decreasing the rotor radius and thickness. Accurate modeling of squeeze-film damping is necessary to predict the dynamic response of an electrical bearing and thus optimize the device geometry and dimensions.

## 5. Experimental Results and Discussion

**Figure 7:**
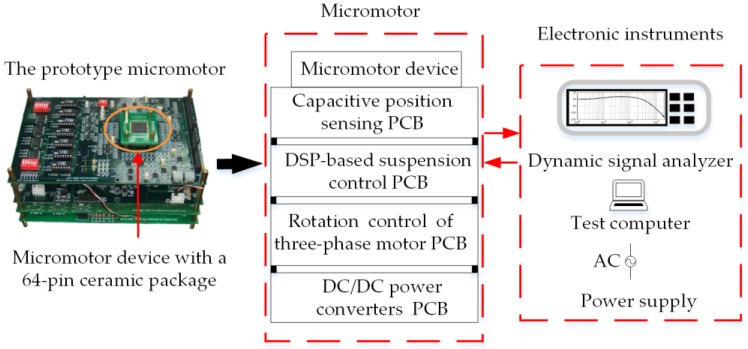
A schematic view of the experimental setup.

Experimental measurements are needed to validate the numerical models used and to ensure that all necessary physical phenomena are taken into account in the damping characterization. Experimental strategies for the measurement of dynamic parameters of the MEMS dampers have been proposed in different studies [17]. One of most popular methods is to extract the quality factor of the mass at resonance. In order to validate the above estimated results by FEM simulation, an electrical measurement method is proposed in Section 2.3 to extract the air-film damping coefficients by experimental frequency responses. The experimental setup, as shown in Figure 7, is composed of four stacked printed circuit boards (PCBs) for five-axis capacitive position sensing, digital bearing control, rotation control of the three-phase micro-motor, and DC/DC power converters, respectively. The micromotor device was wired-bonded to 64-pin ceramic chip package and mounted on the top sensing PCB. The electric bearing system is stabilized by three lag compensators for axial suspension of the rotor in the *z*-, *φ_x_*-, and *φ_y_*-axes and two lag-lead compensators for radial suspension in the *x*- and *y*-axes, respectively. A typical lag-lead compensator is utilized to illustrate the test procedure

(10) $$G_{c}(s)=K_{p}\frac{1+\tau_{g}s}{1+a\tau_{g}s}\cdot \frac{1+\tau_{d}s}{1+b\tau_{d}s},\;a>1,\;b<1$$

where *K*_p_ is the compensator gain, and the subscripts d and g on various time constants indicate the lead and lag part of the compensator, respectively. The controller parameters were selected from simulation results and tuned to enhance the suspension performance by experiments.

Accurate measurement of the damping coefficients produced by air film between the stator and rotor posed several problems. The capacitive position sensing sensitivity *K*_s_ and the gain of the voltage amplifier *K*_a_, need to be calibrated experimentally. Accurate discretization of the suspension controller requires a high sampling frequency. Clearly, the effects of curve-fitting must be addressed to refine the accuracy of the damping measurements.

**Figure 8:**
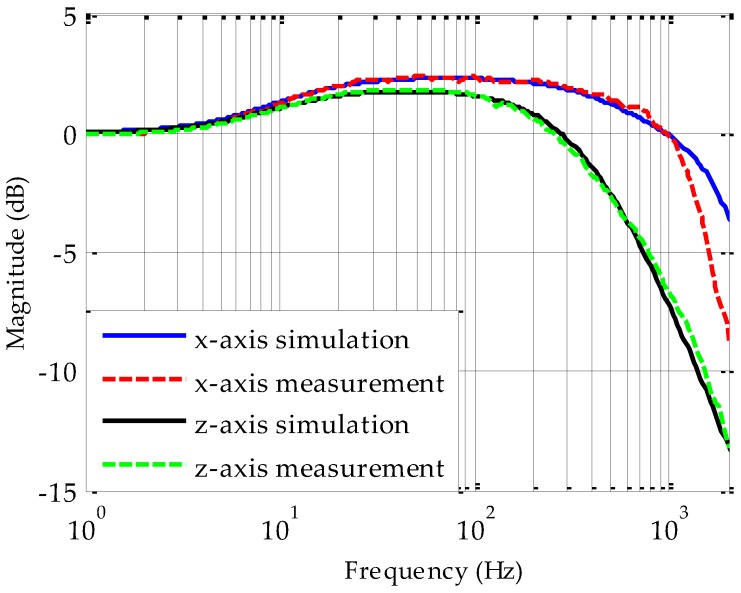
Comparison of two measured and fitted closed-loop frequency responses.

For the experimental results reported in this section, the bearing system was controlled digitally at a sampling rate of 20 kHz. The loop parameters for two translational DOFs are listed in Table 2 for the bearing operated at normal atmosphere pressure. Since the frequency response is critically dependent on the damping coefficients used in the dynamic model of the bearing, the air-film damping coefficients will be extracted from frequency response measurements. A dynamic signal analyzer (Agilent 35670A) was used to output a sweeping-frequency sine signal as the position command input, *x*_c_ and receive the output from the position sensor, *x*_s_. Then the closed-loop frequency response, as defined in Equation (4), can be measured and recorded. Closed-loop frequency responses of the five-axis bearing system are separately measured by means of a sine sweep procedure at frequencies ranging from 1 to 2 kHz. Two experimental frequency responses are presented in Figure 8 as an example, along with their fitted results by simulation. The measurements were performed with a fabricated prototype micromotor operated under atmospheric pressure at room temperature. It is clear that the simulation responses are in good agreement with the experimental curves blow 1 kHz. For higher frequency, the model error due to small time constants neglected in modeling the position sensing circuit, voltage amplifier and digital controller will increase gradually.

Given the measured closed-loop frequency responses shown in Figure 8 and taking the approximate curve fitting of experimental responses as defined in Equation (4), the measured damping coefficients can be determined by tuning the damping coefficient only. The measurement results for five-DOF suspension loops are listed in Table 3, where a single variable Curve Fitting Tool (CFtool) in MATLAB is used to extract these damping coefficients. 

The FEM simulation results and analytical values using the simplified damping models in [22] are also listed in Table 3 for comparison. It is clear that the analytical damping coefficients are quite different from the experimental measurements or FEM-based numerical solutions. For instance, the analytical value of the *x*-axis damping is almost one order of magnitude smaller than other two results. This implies that the constant boundary conditions at ambient pressure applied in solving the linearized Reynolds equations in [22] are not suited to model the air-film damping effect of this sealed device structure. However, the measured air-film damping coefficients agree well with the results of finite element analyzes. For instance, the *z*-axis damping measurement taken from the prototype micromotor shows a relative error of 17.1% lower than the numerical solution. On the basis of the available evidence, this discrepancy is attributed to various dimension errors in fabrication. The simulation results indicate that a damping alteration of 17.1% could result from an air gap error of 9.6% or a rotor size error of 4.6%. Anyway, the experimental results indicate that the FEM-based numerical solution can provide a more precise prediction of air-film damping effect in design and modeling of this micro-motor device where the rotor is suspended in a sealed cavity.

## 6. Conclusions

The FEM-based numerical solutions of the squeeze-film damping and experimental verification of the FEM predication are presented to examine the air-film damping characteristics of the micromotor device in which the mechanical support is replaced by an electrostatic bearing. Note that the air flow coupling effect in the sealed triple-layer device leads to significant cross-axis coupling effect among the five-DOF electric bearing system. As a result, the FEM-based numerical solution is utilized to provide an accurate prediction of air-film damping coefficients which is crucial in device design and modeling of the bearing dynamics. The numerical multiphysics simulations of the air-film damping effect in the five-axis electric bearing are presented, illustrating the change of the squeeze-film damping as a function of the main device dimensions. The measured air-film damping coefficients agree well with the numerical results at atmospheric pressure. It is shown that numerical multiphysics simulation is an effective method to examine the air-film damping effect for complex device geometry and arbitrary boundary condition. The damping coefficients extracted from FEM could be used to accurately model the bearing dynamics which is then used to evaluate the effects of damping on the behavior of the electrostatic bearing system. The accurate damping model will also facilitate successful multi-axis suspension and testing of various micromotor devices. Although the frequency response method for measuring the air-film damping effect prevents precise measurements due to experimental uncertainties and electronic noises, it still represents a significant advance over pervious published research. The solution to the air-film damping measurement problem can be extended to various MEMS sensors and actuators integrated with electrostatic or electromagnetic bearings. 

## Figures and Tables

**Table 1:** Main design parameters of the device.

Description (Unit)	Value
Axial gap *d*_a_ (μm)	6.00
Radial gap *d*_r_ (μm)	6.00
Rotor outer radius *r*_o_ (mm)	2.00
Rotor inner radius *r*_i_ (mm)	1.73
Rotor thickness *h* (μm)	68.00
Mass of the rotor *m* (kg)	4.29 × 10^−7^
Number of rotor poles *N*_r_	14
Rotor poles angle *θ*_r_ (rad)	π/14
Rotor poles outer radius *r_so_* (mm)	1.98
Rotor poles inner radius *r_si_* (mm)	1.88

**Table 2:** Suspension control parameters used in frequency response simulation.

Parameters	*x* Axis	*z* Axis
*K*_v_ (N/V)	2.02 × 10^−6^	4.78 × 10^−6^
*K_i_* (N/m)	4.45	9.32
*K*_s_ (V/m)	0.40 × 10^6^	0.45 × 10^6^
*K*_a_ (V/V)	5.67	4.88
*K*_p_	315	255
*τ*_g_ (s)	0.02	0.02
*aτ*_g_ (s)	1.00	1.00
*τ*_d_ (s)	2.00 × 10^−4^	0.00
*bτ*_d_ (s)	6.00 × 10^−5^	0.00

**Table 3:** Summary of the squeeze-film damping coefficients.

Damping Coefficient	*x*-, *y*-Axes (N·s/m)	*z*-Axis(N·s/m)	*φ_x_*-, *φ_y_*-Axes (N·m /(rad/s))
FEM	2.81 × 10^−3^	2.41 × 10^−2^	4.22 × 10^−8^
Analytical	3.12 × 10^−4^	3.97 × 10^−2^	6.49 × 10^−8^
Experimental	2.30 × 10^−3^	2.00 × 10^−2^	3.40 × 10^−8^
